# Using Connected Vehicle Trajectory Data to Evaluate the Impact of Automated Work Zone Speed Enforcement

**DOI:** 10.3390/s22082885

**Published:** 2022-04-09

**Authors:** Jijo K. Mathew, Howell Li, Hannah Landvater, Darcy M. Bullock

**Affiliations:** 1 Department of Civil Engineering, Purdue University, 207 S Martin Jischke Dr, West Lafayette, IN 47907, USA; howell-li@purdue.edu (H.L.); darcy@purdue.edu (D.M.B.); 2 Project Engineer, RK&K, 651 East Park Drive, Harrisburg, PA 17111, USA; hlandvater@rkk.com

**Keywords:** connected vehicle, trajectory, speeds, automated enforcement

## Abstract

Work zone safety is a high priority for transportation agencies across the United States. Enforcing speed compliance in work zones is an important factor for reducing the frequency and severity of crashes. This paper uses connected vehicle trajectory data to evaluate the impact of automated work zone speed enforcement on three work zones in Pennsylvania and two work zones in Indiana. Analysis was conducted on more than 300 million datapoints from over 71 billion records between April and August 2021. Speed distribution and speed compliance studies with and without automated enforcement were conducted along every tenth of a mile, and the results found that overall speed compliance inside the work zones increased with the presence of enforcement. In the three Pennsylvania work zones analyzed, the proportions of vehicles travelling within the allowable 11 mph tolerance were 63%, 75% and 84%. In contrast, in Indiana, a state with no automated enforcement, the proportions of vehicles travelling within the same 11 mph tolerance were found to be 25% and 50%. Shorter work zones (less than 3 miles) were associated with better compliance than longer work zones. Spatial analysis also found that speeds rebounded within 1–2 miles after leaving the enforcement location.

## 1. Introduction

Ensuring compliance with work zone speeds is a critical objective for transportation agencies and partners across the United States. Every year there are nearly 800 fatalities and more than 120,000 work zone related crashes [1]. Enforcing speed compliance is one way to improve work zone safety and reduce crashes. Although several studies have found law enforcement to have the largest impact on speed compliance [2,3,4,5], this solution is not scalable, especially due to the lack of both staffing and resources. Moreover, enforcement activities in work zones can sometimes be dangerous for both traveling motorists and enforcement officers. In the past decade, automated speed enforcement programs using both radar and camera-based technology have gained popularity. Several studies have shown that automated enforcement programs can significantly reduce work zone speeds [6,7,8]; however, most of them were limited to assessing the localized impact on speeds. The presence of equipment and/or personnel when speeds are collected can also bias the data. Connected vehicle (CV) trajectory data now provide an opportunity to perform a comprehensive analysis of work zone speeds over extended periods without introducing any sampling bias. The objective of this paper is to describe a methodology using CV trajectory data to inform agencies on the operation of automated enforcement. 

This paper is organized as follows: the first section introduces the problem statement and objective of this study; this is followed by an enhanced literature review and identification of research gaps. The third section provides a summary of the Automated Work Zone Speed Enforcement program in the Commonwealth of Pennsylvania, followed by the study scope and objectives. The fifth and sixth sections describe the connected vehicle trajectory data and enforcement data used in this study. The following sections present data analysis and speed compliance results during the presence and absence of automated enforcement. This is followed by a presentation of the findings regarding the impact of work zone length on speed compliance and a comparison of automated work zones in Pennsylvania with work zones in Indiana, where no automated enforcement is employed. The final section provides a summary of this research along with potential future work opportunities.

## 2. Literature Review

Speeding is a major factor that influences the severity and frequency of crashes in work zones [9,10,11,12]. Agencies have adopted several measures to control work zone speeding, including but not limited to, reduced speed limit signs [13], radar-based speed feedback displays [13,14,15], variable message signs [14,16] and transverse rumble strips [17]. Enforcement measures such as the presence of law enforcement [2,14] and cameras [13] have also been instrumental in reducing work zone speeds.

Fontaine et al. conducted a feasibility study of real-time remote speed enforcement for work zones and found that it may overcome many of the legislative barriers that prevent the use of automated enforcement and also provide a safety benefit to law enforcement officers [18]. In 2004, Illinois became the first state to pass legislation that allowed the use of automated speed photo-radar enforcement (SPE) in work zones. Subsequent studies on Illinois work zones found that SPE was effective in reducing speeds of cars and heavy vehicles during both free-flow and general traffic stream conditions [6,7,8,19]. The average reductions in speeds were found to be between 4 and 8 mph for cars and between 3 and 7 mph for heavy vehicles. The reduction in speeding at 1.5 miles downstream of the enforcement was found to vary between 0% and 44%. Studies in Arizona revealed that automated enforcement using a fixed camera on Arizona State Route 101 reduced speeds by 9 mph and the estimated total number of target crashes by 44–54% [20]. Studies by other agencies including Washington DOT [21], Oregon DOT [22] and Maryland DOT [23] also found significant speed reductions with the use of automated enforcement. In Maryland, where the program has been deployed at nearly 100 work zones since 2010, the number of vehicles exceeding the speed limit tolerance (12 mph above speed limit) has been reduced by nearly 90% since its inception.

Although the localized impact of automated enforcement is well documented, there are very limited studies that have looked at the spatial and temporal compliance of speeds across the work zone. Franz et al. conducted a study using tube counters and microwave sensors to understand the spatial (2 miles upstream and downstream) and temporal speeding effects, and found that speeds tend to reduce at enforcement locations but increase after the enforcement zone [24]. Temporal analysis showed a general reduction in aggressive driving during the enforcement period with more stable spatial speeding distribution. Wasson et al. used Bluetooth probe data to study the spatial and temporal impact of overall speeding during enforcement activity on a 12 mi work zone in Indiana [25]. Their results showed that space mean speeds dropped by approximately 5 mph throughout the work zone during enforcement and increased within 30 min after the enforcement detail ended. 

CV trajectory data, which provides a large sample that extends both spatially and temporally across the work zone, enables researchers to perform a holistic analysis of the speeding patterns inside the work zone. Few studies have used this data to understand speed compliance in work zones. Mathew et al. studied the spatial and temporal effect of speed feedback display signs and posted speed limit signs on a 15 mi work zone in Indiana [26]. While posted speed limit transitions did not have any impact on speed reductions, the speed feedback display sign saw a maximum reduction in median speeds by 5 mph. The overall reach of the data enabled the researchers to discover that geometric constraints such as lane closures and tight shoulders had the most impact on speed reductions in the work zone.

As seen, several studies have evaluated the performance of automated enforcement; however, the evaluation was mostly carried out at a localized level—either at the location of the enforcement or a few miles upstream or downstream of the enforcement. Although automated enforcement was found to improve the speed compliance in such cases, it is also necessary to understand the impact across the entire length of the work zone. The ubiquity of connected vehicle data now provides an opportunity to perform a holistic analysis without the need for any significant infrastructure investments. The objective of this paper is to describe the methodology for using CV trajectory data to characterize both the spatial and temporal effects of automated enforcement on driver behavior throughout the length of the work zone.

## 3. Automated Speed Enforcement in Pennsylvania

**Figure 1:**
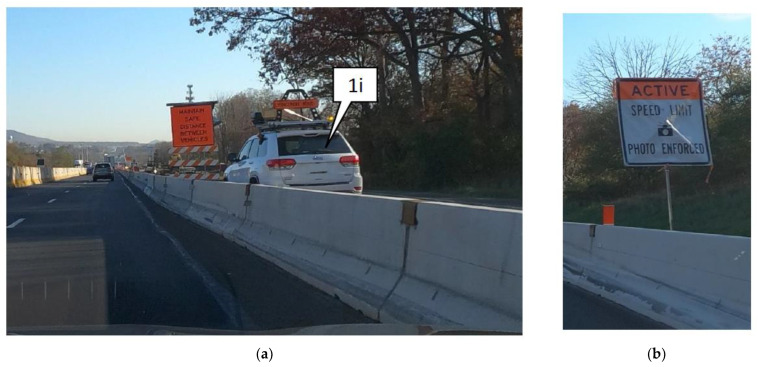
Automated speed enforcement field unit and warning signs. (**a**) Field unit with radar equipment and roof mounted cameras. (**b**) Speed enforcement warning sign. Callout 1i shows the field unit on I-78 work zone in I-78, Pennsylvania.

In 2020, there were nearly 1300 work zone crashes in Pennsylvania, which resulted in over 800 injuries and 15 fatalities [27]. The automated work zone speed enforcement (AWZSE) program in Pennsylvania was enacted into law by Act 86 (2018), which authorizes automated speed enforcement in active work zones [28]. This program is jointly supported by the Pennsylvania Department of Transportation, the Pennsylvania Turnpike Commission and the Pennsylvania State Police with the goal of promoting work zone safety by reducing speeds and improving driver behavior. A field unit is deployed (Figure 1a(1i)) in the work zone (typically for 8 h per day), which uses both radar and roof-mounted cameras to capture vehicle speeds. During active enforcement, advance warning signs (Figure 1b) are also placed at 500 ft and 1000 ft ahead of the field unit to alert the incoming motorists. Violations are issued for speeds exceeding a tolerance of 11 mph over the speed limit. The first offense is treated as a zero-first violation and fines are issued for repeat violators, i.e., for the second and any subsequent offenses.

Statewide automated enforcement began in March 2020 but was halted due to the pandemic and then resumed at critical and emergency work zones in April 2020. In 2020, there were over 2000 deployments, which resulted in more than 219,000 violations and roughly $1.7 M of fines from nearly 12% repeat offenders. Preliminary results showed that speeds dropped in the work zones, with a 16.6% reduction in the percentage of vehicles travelling over the speed limit and a 43.6% reduction in the percentage of vehicles over the speed limit tolerance [28].

## 4. Scope and Objective

The scope of this paper is to present a methodology for using CV trajectory data to characterize the speed in three work zones in Pennsylvania that had automated speed enforcement deployed between April and August 2021. This includes the following:

- An evaluation of speed limit compliance during the presence and absence of automated enforcement;
- A comparison of speed compliance on short versus long work zones;
- Performing longitudinal analysis of the speed variation across the length of the work zone;
- A comparison of speed compliance with work zones without automated enforcement in Indiana.

## 5. Work Zones in Pennsylvania with Automated Speed Enforcement

**Figure 2:**
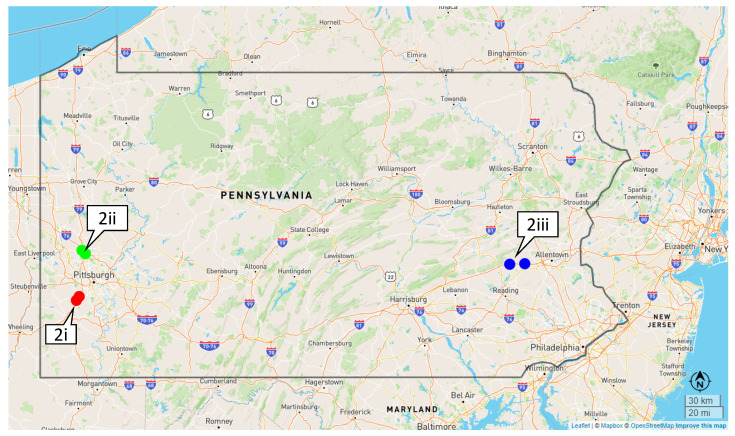
Study work zones in PA with active automated speed enforcement. Callout 2i, 2ii and 2iii shows the I-79, I-76 and I-78 work zones, respectively.

Three work zones with active automated speed enforcement between April and August 2021 were selected for this study (Figure 2). The first work zone is located south of Pittsburgh on I-79 south (S), between mile markers (MM) 51 and 48, with a work zone speed limit of 45 mph (Figure 2(2i)). The second work zone is located north of Pittsburgh on the Pennsylvania Turnpike I-76 W between MM 31 and 28 (Figure 2(2ii)). The speed limit in this work zone is 55 mph. The final work zone is a 10-mile section between MM 45 and 35 on I-78 W. The speed limit in this work zone, located west of Allentown (Figure 2(2iii)), is 50 mph. All three work zones underwent reconstruction activities and generally maintained two travel lanes within barrier protection while enforcement was present. Detailed logs with durations and locations of active enforcement on each day were also available. Automated enforcement was not deployed during adverse weather conditions such as heavy rains and thunderstorms. Although previous studies have used probe vehicle data to capture speed variations during rain events [29], this paper does not consider the impact of weather.

## 6. Anonymized Connected Vehicle Trajectory Data

**Figure 3:**
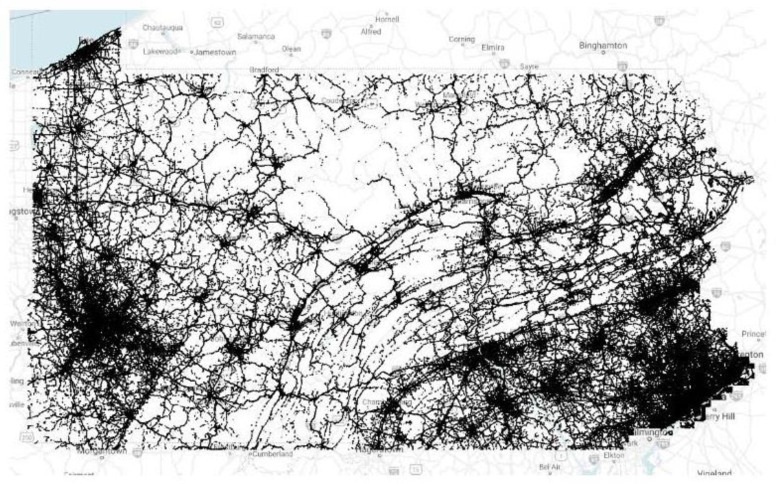
CV trajectory data points during an hour in PA.

The CV data used in this study were obtained from a third-party commercial data vendor that partners with original equipment manufacturers (OEM) to provide anonymized vehicle trajectories. Each trajectory consists of a series of waypoints with a reporting frequency of 1–3 s and a spatial fidelity of 3 m. Each waypoint consists of unique trajectory identifiers, geographic coordinates, timestamps, speeds, headings and ignition statuses for passenger cars. Previous studies have indicated that these CV data represent approximately 3–5% of the total vehicles operating on interstates [30]. For the study period between April and August 2021, there were approximately 71 billion CV records available in Pennsylvania. To portray the extent and coverage of this data, Figure 3 shows a map of nearly 35 million CV records generated during the noon hour on one day in Pennsylvania. During this study, over 322 million CV records from nearly 538,000 unique trips were extracted across the three work zones of interest. I-79, I-78 and I-76 generated roughly 162, 100 and 60 million records in both directions, respectively. I-79 S, I-78 W and I-76 W returned 87, 50 and 30 million records, respectively.

### Spatial Referencing CV Data to Roadway Mile Markers

**Figure 4:**
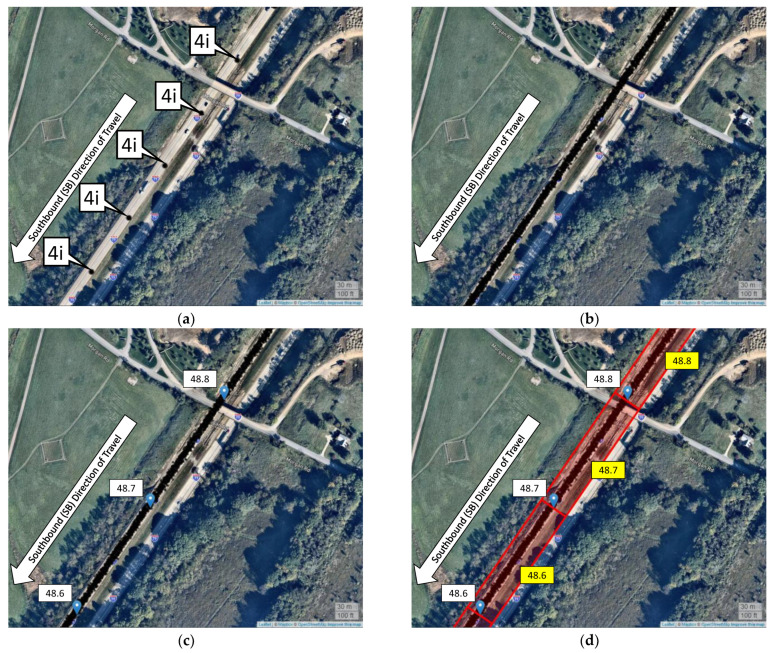
Geospatial joining of connected vehicle trajectories to roadway mile markers. (**a**) Trajectory data every 3 s from one CV. (**b**) All CV trajectories. (**c**) Overlaying interstate mile markers. (**d**) Geospatial joining of CV trajectory points to interstate mile marker polygons. Callout 4i shows the datapoints from one CV.

To perform spatial analysis, it is necessary to transform the data relative to the mile markers (MM) or mile posts on the roadway. This is accomplished by conducting a geospatial join of CV data and interstate mile markers. Figure 4a shows the data points (Figure 4(4i)) every three seconds from one CV travelling on I-79 S. Figure 4b shows data points from multiple trajectories on this section. The mile markers occurring every tenth of a mile (MM 48.8, 48.7 and 48.6) are then overlaid and connected by line segments (Figure 4c). A spatial polygon is developed by adding a two-sided buffer to this line segment connecting the mile markers, and the CV data points within this polygon are joined to the mile markers (Figure 4d). For example, all the CV data points between MM 48.6 and 48.7 are assigned a spatial reference of MM 48.6. In addition to this spatial joining, a heading filter (+/−10° of the roadway line segment heading) is also applied to the CV data to remove directional outliers.

## 7. Impact of Automated Enforcement across Work Zones

Analysis was limited to periods between 6 A.M. and 6 P.M. to remove any potential bias outside enforcement hours. Speeds below 25 mph were also discarded to exclude congestion and queuing impacts. In addition, speeds above 120 mph were treated as outliers and excluded from the analysis.

### 7.1. Interquartile Range (IQR) Plots

**Figure 5:**
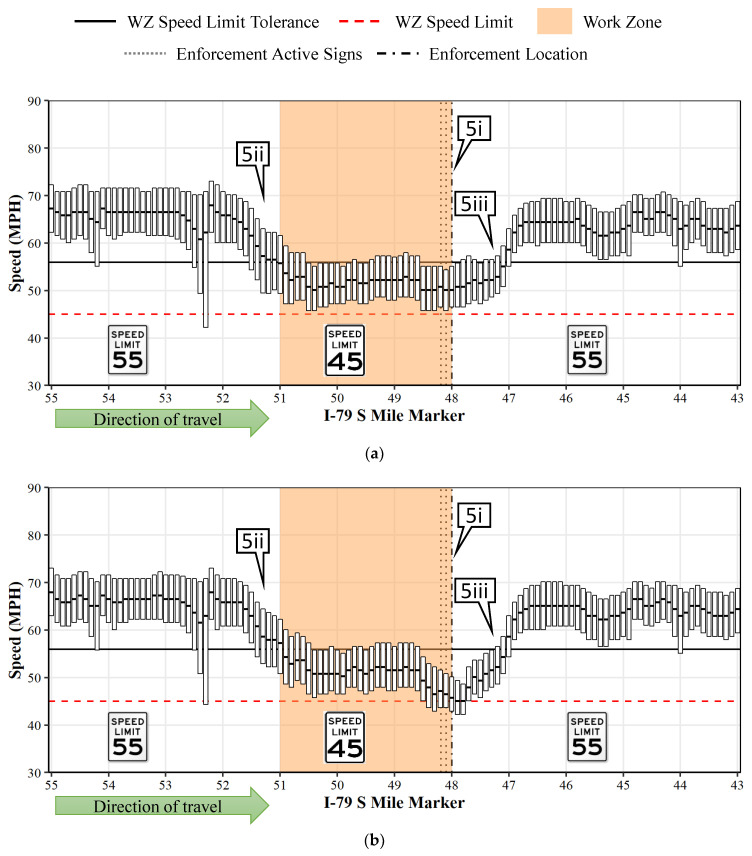
IQR plots comparing impact of enforcement on I-79 SB work zone. (**a**) Enforcement absent. (**b**) Enforcement present. Callout 5i shows the enforcement location; 5ii highlights the reduction in speed before entering the work zone; and 5iii highlights the increase in speed after exiting the work zone.

Figure 5 illustrates an IQR plot of speeds every 0.1 miles along the study section between MM 55 and 43 on I-79 S. The x-axis indicates the mile markers along the direction of travel, and the y-axis shows the speeds. The normal speed limit on this section of I-79 is 55 mph. The bottom bar of the interquartile plots shows the 25th percentile, the top bar shows the 75th percentile and the middle bar shows the median speed. The tan backfill highlights the work zone extents, the horizontal dotted red line represents the work zone speed limit (45 mph) and the horizontal black solid line represents the 11-mph speed limit tolerance (56 mph). The vertical dot-dash line shows the location of automated enforcement (at MM 48) and the two dotted lines before it shows the location of the warning signs (Figure 1b).

Figure 5a depicts the variation in speeds when automated enforcement was absent (at MM 48), and Figure 5b depicts a similar graphic when enforcement was present (at MM 48). When the two figures are compared, there is a noteworthy drop in speeds on Figure 5b around the enforcement location (Figure 5(5i)). On closer examination, the drop in speeds begin a few tenths of a mile ahead of the warning signs indicating a strong speed limit compliance during the presence of enforcement. The speeds further drop when the motorists see the enforcement vehicle in their sight, and the drop in speeds continues for approximately 0.2 miles past the enforcement vehicle. It is also interesting to note that, irrespective of the enforcement activity, nearly 75% of all the speeds inside the work zone are within the speed limit tolerance.

**Figure 6:**
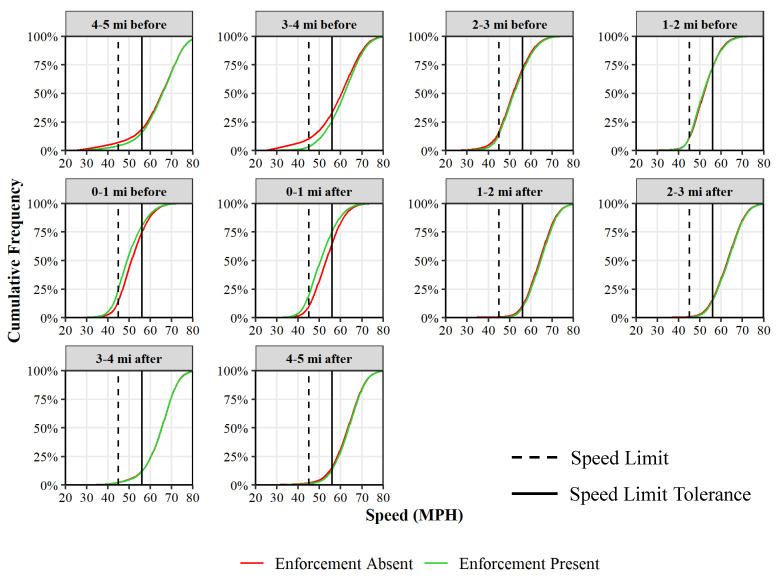
Speed compliance with respect to enforcement location on I-79 S work zone.

Figure 5 also provides an opportunity to understand the speed patterns before entering and after exiting the work zone. The reduction in speeds begin roughly 1 mile before entering the work zone (Figure 5(5ii)). Similarly, the speeds climb back up to 55 mph within a mile after exiting the work zone (Figure 5(5iii)). This is further corroborated in Figure 6, which compares the cumulative frequency of the speeds during the presence (green) and absence (red) of enforcement across 1-mile sections before and after the location of the enforcement. For the 1-mile stretch just before and after the enforcement location, there is a shift in the green curve (enforcement present) towards the left signaling a reduction in speeds. For all the other sections, the two curves overlay each other, indicating similar vehicle speeds.

A nonparametric Kolmogorov–Smirnov test (K-S test) is also conducted for detecting the horizontal differences between the two distributions [31]. The D-statistic shows the maximum vertical distance between the two cumulative frequency diagrams on Figure 6. Table 1 shows the results from the K–S test at every 1-mile section before and after the location of enforcement. Results show that the distributions during the presence and absence of enforcement are statistically significant at all locations (at a 99% confidence level), with the maximum separation observed within 1 mile of the enforcement location. 

Although K–S test show statistically significant differences with and without enforcement at multiple locations, the more important takeaway from Figure 6 is that the upper tails (high speeds) are quite close and demonstrate the impact of reducing high speeds in work zones, even when the automated enforcement is not present.

### 7.2. Speed Compliance Proportion

**Figure 7:**
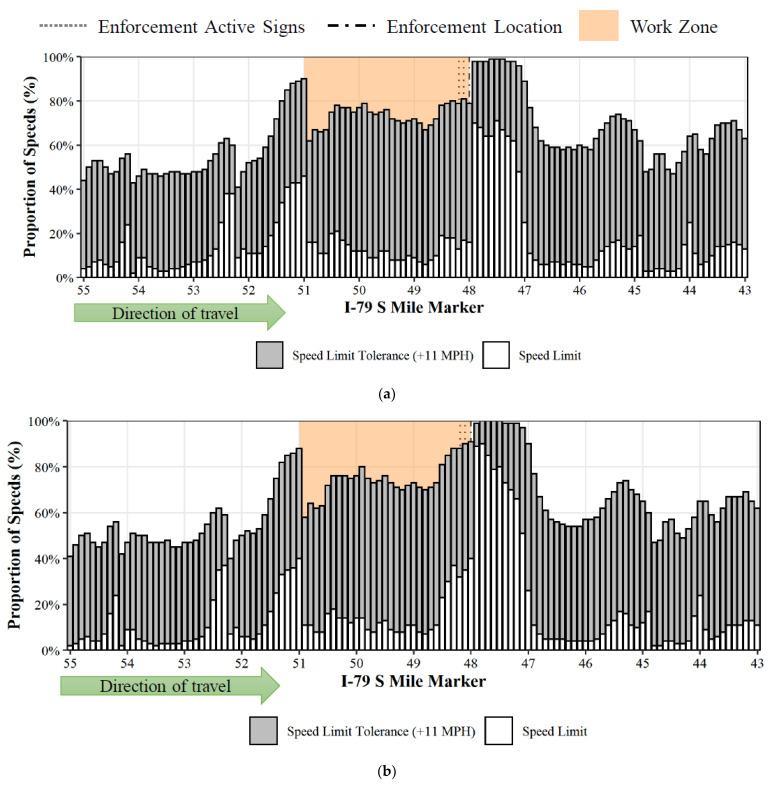
Compliance with speed limit and within speed limit tolerance, with and without enforcement. (**a**) Enforcement absent. (**b**) Enforcement Present.

These data also provide an opportunity to conduct a longitudinal analysis of speed compliance over the entire length of the work zone. Figure 7 illustrates the speed compliance both with the speed limit and within the speed limit tolerance across the I-79 S work zone, during the absence (Figure 7a) and presence (Figure 7b) of enforcement at MM 48.0. In general, irrespective of the enforcement, the average speed limit compliance (inside the work zone) was roughly 15%, whereas compliance within the speed limit tolerance was nearly 75%. During active enforcement, the average compliance with the speed limit and within the speed limit tolerance increased by roughly 3% and more than 1%, respectively, compared to periods without enforcement.

Table 2 presents a summary of the average compliance over the three work zones during the presence and absence of automated enforcement. The 3-mile I-76 W work zone recorded the highest compliance, with more than 25% under the speed limit and nearly 85% under the speed limit tolerance. However, the presence of enforcement was not found to have a major impact on the speed compliance. The I-78 W work zone was found to have the least compliance—a little above 10% under speed limit and close to 60% under speed limit tolerance. This could be due to the greater length of this work zone, which stretches over 10 miles. 

## 8. Impact of Work Zone Length

**Figure 8:**
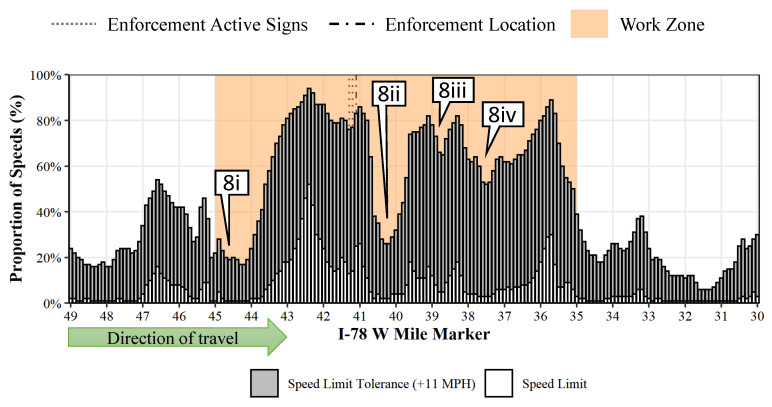
Compliance with speed limit and within speed limit tolerance on 10 mi I-78 W work zone. Callouts 8i-8iv highlights the speed fluctuations.

Although there are no standard definitions for classifying work zones based on length, in this study we consider work zones less than 3 miles to be short work zones and those greater than 3 miles to be long work zones. Among the three work zones, the two shorter ones (I-79 S and I-76 W) were found to have better speed compliance than the longer I-78 W work zone. As seen before in Figure 7b, there are less fluctuations in speed compliance within the 3-mile I-79 S work zone. The speed compliance is uniform for most of the work zone, except towards the end where the compliance increases due to the presence of enforcement. Figure 8 shows the proportion of speed compliance over the 10-mile I-78 W work zone. In contrast, there are few considerable fluctuations (Figure 8(8i–8iv)) on the longer work zone—potentially areas free of work activity and geometric constraints such as lane reductions. The level of compliance within the speed limit tolerance even drops to less than 25% in a few of these zones (Figure 8(8i,8ii)). It is common for agencies to combine two or more work zones separated by a short distance (less than a mile or two) into a single work zone; however, our findings suggest that this could lead to several speed fluctuations and, more importantly, a significant reduction in speed compliance.

## 9. Comparison with Work Zones without Automated Enforcement

**Figure 9:**
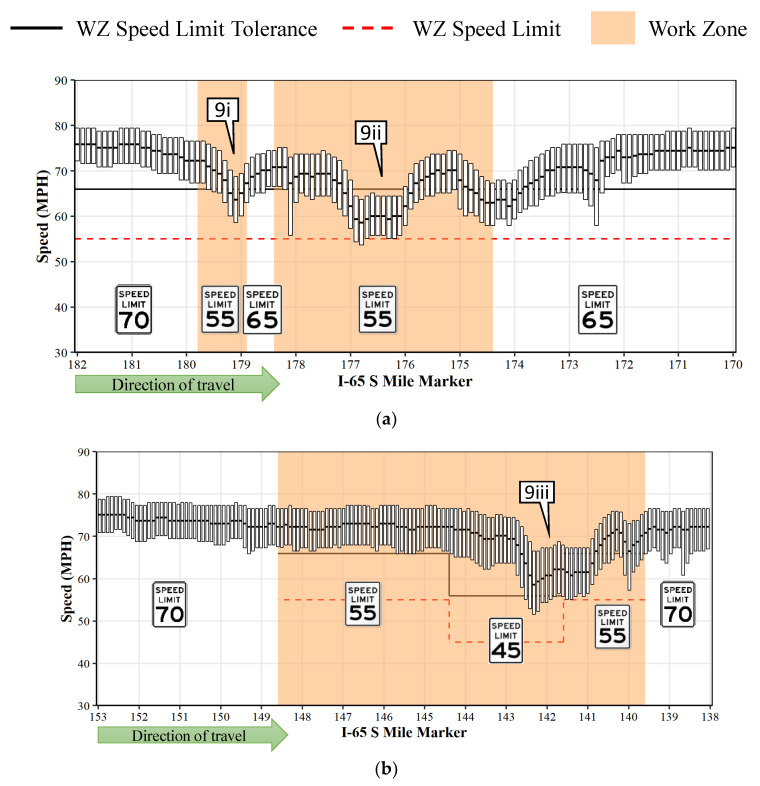
IQR speed plots for non-enforcement work zones. (**a**) I-65 Sa WZ MM 182 to MM 170, IN during July 2021. (**b**) I-65 Sb WZ MM 153 to MM 138, IN during May 2021. Callouts 9i-9iii shows the speed reductions due to geometric constraints.

Figure 9 illustrates the IQR plots (similar to Figure 5) for two work zones without automated enforcement on I-65 S in Indiana. Figure 9a represents the work zone from MM 182 to MM 170 during the month of July 2021 (herein referred to as I-65 Sa) and Figure 9b represents the work zone from MM 153 to MM 138 during May 2021 (referred to as I-65 Sb). The speed limits (dotted red horizontal line) on both work zones transition from 70 to 55 mph, with I-65 Sb also having a section with a 45 mph limit. The speed limit tolerance (solid black horizontal line) is offset by 11 mph from the speed limit. For I-65 Sb, the IQR plots occlude the speed limit tolerance line when the speeds go back to 55 mph at MM 141.6 (Figure 9b). Both work zones underwent reconstruction activities with partial lane reductions.

As indicated earlier, neither of these work zones had automated enforcement, and only occasional enforcement by officers in marked cruisers due to narrow shoulders that provided a challenging environment for safely monitoring and/or stopping motorists. In general, there were only a few sections where more than 75% of the speeds were within the speed limit tolerance (Figure 9(9i–9iii)). Further investigations revealed that geometric constraints such as lane reductions (Figure 9(9i,9ii)) and narrow lanes without any shoulders (Figure 9(9iii)) resulted in these speed drops [26]. On I-65 Sb, almost all of the median speeds were roughly 20 mph over the speed limit, except the zone with narrow shoulder shown by callout 9iii. The average compliance with the speed limit across the work zone was found to be less than 11% for I-65 Sa and less than 5% for I-65 Sb (Table 3). Only half of the analyzed speeds were within the speed limit tolerance on I-65 Sa, whereas for I-65 Sb, less than a quarter of the speeds were within the speed limit tolerance.

**Figure 10:**
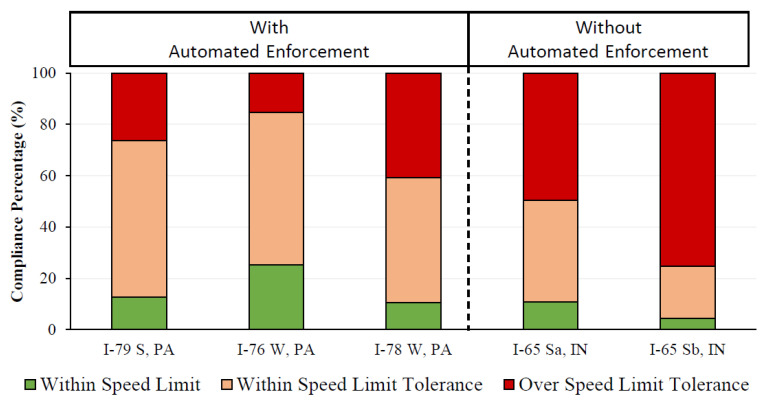
Speed compliance comparison for work zones with and without enforcement.

Figure 10 provides an overall comparison of the speed compliance across the PA work zones with automated enforcement and IN work zones without automated enforcement. In general, the work zones with automated enforcement performed well, with nearly 12–60% better compliance. The I-78 W (PA) and I-65 Sa (IN) work zones have comparable speed compliance, possibly due to their longer extents (over 10 miles in length).

## 10. Summary and Conclusions

This study analyzed over 322 million connected vehicle records between April and August 2021 to study the spatial and temporal impact of the automated work zone speed enforcement program on three work zones in Pennsylvania. Compliance with the speed limit and within a speed limit tolerance of 11 mph over the speed limit were evaluated. The graphical visualizations provide an overall understanding of the speed variations (Figure 5) and compliance (Figure 6) throughout the length of the work zone as well as before entering and after leaving the work zone. Reductions in speeds were found to occur within a mile before entering the work zone. Similarly, speeds went back up within a mile after passing the work zone.

During the absence of enforcement, the average compliance with work zone speed limits ranged from 10–25% and compliance within the speed limit tolerance ranged from 59–84%. During enforcement, the average compliance with the work zone speed limit ranged from roughly 11–27% and compliance within the speed limit tolerance ranged from 62–84% (Table 2). Short work zones (3 miles or less) were found to have better speed limit compliance (Figure 8) than longer work zones (10 miles).

This study also compared and evaluated the speed limit compliance on two work zones in Indiana without automated enforcement. Compliance with the speed limit and within the speed limit tolerance was estimated to be only around 4–11% and 25–50%, respectively (Table 3, Figure 10).

The connected vehicle data used in this study not only remove the need for on-site data collection, but also provides high-fidelity samples of vehicular speeds at 1–3 s intervals. Although the qualitative analysis presented in this study highlights discernible variations in speeding patterns during the presence and absence of automated enforcement, there could be several other factors that impact the driving behavior of motorists inside a work zone. Future research will include a comprehensive statistical analysis and econometric modeling that captures work zone geometry, work zone type (short term/long term), time of day (day/night), weather and vehicle type (passenger car/truck) to understand the significant factors that impact driving behavior inside the work zone.

The analysis and visualizations presented in this study highlight the reach and scalability of this big data to facilitate a comprehensive analysis of driving behavior and speeding compliance in the work zones. Although this is a sample dataset of all the vehicles in the traffic stream, the methodologies and framework provide a complete understanding of the speeding patterns across the entire length of the work zone, which agencies can use for better planning and resource allocations.

## Figures and Tables

**Table 1:** Kolmogorov–Smirnov test showing the distributions during the presence and absence of enforcement.

Location	D-Statistic	*p*-Value
4–5 mi before	0.033 *	<0.001
3–4 mi before	0.075 *	<0.001
2–3 mi before	0.028 *	<0.001
1–2 mi before	0.024 *	<0.001
0–1 mi before	0.129 *	<0.001
0–1 mi after	0.146 *	<0.001
1–2 mi after	0.03 *	<0.001
2–3 mi after	0.018 *	<0.001
3–4 mi after	0.004 *	0.007
4–5 mi after	0.025 *	<0.001

* Significant at 99% confidence level.

**Table 2:** Summary of average speed compliance inside the work zone with and without enforcement.

WZ	Length (mi)	WZ Speed Limit (mph)	WZ Speed Limit Tolerance (mph)	Compliance with WZ Speed Limit		Compliance within WZ Speed Limit Tolerance	
				Enf. Absent	Enf. Present	Enf. Absent	Enf. Present
I-79 S	3	45	56	12.7%	15.4% (↑)	73.7%	74.8% (↑)
I-78 W	10	50	61	10.6%	11.5% (↑)	59.3%	62.6% (↑)
I-76 W	3	55	66	25.4%	26.9% (↑)	84.6%	84.3% (↓)

**Table 3:** Summary of average speed compliance on IN work zones without automated enforcement.

WZ	Length (mi)	WZ Speed Limit (mph)	WZ Speed Limit Tolerance (mph)	Compliance with WZ Speed Limit	Compliance within WZ Speed Limit Tolerance
I-65 Sa	5	55	66	10.8%	50.3%
I-65 Sb	9	45/55	56/66	4.3%	24.7%

## Data Availability

Not applicable.
